# Simultaneous multislice imaging of the heart using multiband balanced SSFP with blipped‐CAIPI

**DOI:** 10.1002/mrm.28086

**Published:** 2019-11-20

**Authors:** Anthony N. Price, Lucilio Cordero‐Grande, Shaihan J. Malik, Joseph V. Hajnal

**Affiliations:** ^1^ School of Biomedical Engineering and Imaging Sciences King's College London London United Kingdom

**Keywords:** balanced SSFP, banding artefacts, blipped‐CAIPI, cine CMR, multiband, simultaneous multislice

## Abstract

**Purpose:**

In this work, we explore the use of multiband (MB) balanced steady‐state free precession (bSSFP) with blipped‐controlled aliasing in parallel imaging (CAIPI), which avoids the issues of altered frequency response associated with RF phase cycling, and show its application to accelerating cardiac cine imaging.

**Methods:**

Blipped and RF‐cycled CAIPI were implemented into a retrospective‐gated segmented cine multiband bSSFP sequence. The 2 methods were compared at 3T using MB2 to demonstrate the effect on frequency response. Further data (4 subjects) were acquired at both 1.5T and 3T collecting 12‐slice short axis stacks using blipped‐CAIPI with MB acceleration factors of 1–4. The impact on SNR and contrast was evaluated along with g‐factors at different accelerations.

**Results:**

Data acquired with blipped‐CAIPI multiband bSSFP up to factor 4 yielded functional cine data with good SNR and contrast, while reliably keeping dark‐band artefacts clear of the heart at 1.5T. SAR limits the maximum MB acceleration, particularly at 3T, where minimum TR increase is problematic and leakage artefacts are more prevalent. Mean g‐factors across the heart were measured at 1.00, 1.06, and 1.12 for MB2–MB4, whereas blood‐pool SNR measures (end‐diastole) decreased by 11.8, 21.5, and 36.9%; ultimately LV‐myocardium CNR remained sufficient at 1.5T with values ranging: 15.6, 13.4, 11.9, and 9.6 (MB1–MB4).

**Conclusion:**

Blipped‐CAIPI multiband bSSFP can be used in cardiovascular applications without affecting the frequency response because of controlled aliasing and can be readily incorporated into segmented cine acquisitions without adding any additional constraints because of phase cycling requirements. The method was used to collect full ventricular coverage within a single breath‐hold.

## 1 INTRODUCTION

Simultaneous multislice imaging has seen rapid uptake in recent years, where it is becoming a routine method in neuroimaging to accelerate 2D multislice echo‐planar acquistions.^1^ The method uses multiband (MB) radiofrequency pulses to excite more than 1 slice simultaneously with reconstruction of superimposed slices using parallel imaging methods.^2^ Slice acceleration has also been applied in cardiovascular MR (CMR) applications, primarily using spoiled gradient echo (SPGR) acquisitions,^3^, ^4^, ^5^ however, combining with balanced steady‐state free precession (bSSFP),^6^ which has become the gold‐standard choice for functional cine imaging,^7^ is more challenging.

A key development in the practicality of multiband acceleration was the introduction of controlled aliasing in parallel imaging results in higher acceleration (CAIPIRINHA)^8^ whereby the FOV of neighboring slices is shifted to impart more diversity between the coil sensitivities of the superimposed slices, dramatically reducing the g‐factor^9^ and improving the quality of image reconstruction. The technique works by adding a distinct phase pattern to consecutive lines of k‐space on each slice. Originally, this was achieved by cycling between multiple RF pulses that have the necessary differential slice dependent phase shifts.^8^ However, in bSSFP, the RF phase is already cycled to center the pass band of the frequency response on‐resonance, so any additional phase cycling required for CAIPIRINHA also shifts the frequency response, and therefore the location of dark‐band artefacts. This is particularly problematic in cardiac cine imaging where care needs to be taken to avoid bands in the blood pool,^10^, ^11^ with the issue becoming more challenging at higher field strengths where precise localized shimming is already essential.^12^, ^13^

The first practical implementation of multiband CAIPIRINHA with bSSFP in CMR was demonstrated by Stab et al^14^ using an RF cycling scheme that distributed the FOV shift across both slices in MB2 while maintaining bSSFP phase cycling requirements and minimising g‐factor. However, because of the reduced effective pass band, the method is limited to lower field strengths and MB factors. A recent advancement of the technique incorporates an unbalancing of the slice re‐phasing gradient to adjust the local Larmor frequency at each slice location, alleviating the limitations on MB factor and largely regaining the original effective pass band, although the frequency response is now partly influenced by the MB gap to slice thickness ratio.^15^

An alternative approach to CAIPIRINHA with RF phase cycling is to use gradient blips (blipped‐CAIPI) to impart spatially dependent phases to all the excited slices. This is the only practical option for single‐shot methods such as echo‐planar imaging,^16^ but can equally be used for SPGR and has been described in a patent for bSSFP^17^ sequences, but has not to our knowledge been explored or characterised. There are several convincing arguments for adopting this technique for MB bSSFP in cardiac imaging. Primarily, the original phase cycling of the RF is maintained, avoiding the issues caused by shifting the frequency response with controlled aliasing. This enables MB to be used with bSSFP at higher field strength without increasing the already restrictive problems caused by off‐resonance artefacts in cine imaging, beyond that associated with unavoidable TR increases because of the higher energy in MB pulses that increase SAR.

Furthermore, the use of optimal phase combination^18^ to limit peak RF field (*B*_1_), and therefore pulse length, is simple to apply with only a single multiband RF pulse being necessary. RF phase‐cycled CAIPIRINHA requires an increasing number of sub pulses with higher MB and shift factors. For example, a cycle of 4, 6, and 8 pulses would be required for MB = 2, 3, and 4 acquisitions, respectively, assuming the shift pattern is equally distributed across all MB slices. The requirement to complete this cycle also introduces restrictions on the number of cardiac phases and/or k‐space lines that can be acquired in each segment of the standard cine sequence. Ordinarily the number of (in‐plane) phase‐encode lines (k_y_) acquired per segment is set simply by dividing the anticipated R‐R interval by the TR and the desired number of cardiac phases. Acquiring multiple phase‐encode lines (aka TFE factor) in each cardiac phase window is a potent acceleration technique that can be freely used without constraint using blipped‐CAIPI MB bSSFP, but forces a commensurate relationship between TFE factor and 2xMB factor with RF phase‐cycled CAIPIRINHA.

Finally, the optimum choice of aliasing pattern that best suites the MB factor and coil geometry to minimise g‐factor is easily applied with gradient blipping without changing the RF pulse. For example, with MB4, a relative shift of FOV/3 between consecutive slices may improve slice unfolding compared to distributing shifts across the full MB FOV. Similarly, different combinations of shift patterns could easily be temporally interleaved for self‐calibration or flexible spatio‐temporal sampling trade‐offs, if prospective gating or free running acquisitions are used.

Here, we present data from a full implementation of multiband with blipped‐CAIPI into a bSSFP retrospectively gated segmented cine sequence typically used to assess cardiac function. A potential disadvantage of blipped‐CAIPI bSSFP over RF phase cycling methods could be increased eddy currents,^19^ therefore this was also tested for in phantoms. Controlled aliasing using gradient blips preserves the bSSFP frequency response without restriction on acquisition parameters or optimal phase to control peak *B*_1_. Data has been acquired at both 1.5T and 3T on healthy adult volunteers with slice accelerations of up to MB4, which can enable full ventricular slice coverage within a single extended breath‐hold, adding the potential to improve functional analysis by avoiding misalignment of slices. Image reconstruction was performed using parallel imaging methods, independently slice‐by‐slice without regularization, therefore fully preserving the temporal fidelity and acquired resolution of dynamic structures through the cardiac cycle.

## 2 THEORY

A standard segmented cine bSSFP sequence was modified to allow on‐the‐fly modulation of the RF pulse to generate the desired MB factor and slice separation. Conventional phase‐cycling of π is applied to the MB pulse independently of k‐space position or cardiac phase. To achieve the relative FOV shift between slices, an additional cyclic gradient increment along z of *A_blip_* = 2π/(γ × *z_gap_* × *f_shif_*_t_) is required between successive TR periods (assuming k_y_ is also stepping linearly), where *z_gap_* is the separation between consecutive MB slices and FOV/*f_shift_* is the desired shift between neighboring slices, with *f_shift_* = 2, 3, 4 used in this study, and γ the gyromagnetic ratio. The gradient blipping pattern is also balanced to center the point spread function relative to the slice encoding direction in k‐space (k_z_). This means that rather than toggle. for example. between 0 and π for MB2, a ±π/2 blip pattern is used, and similarly for MB3 (FOV/3 shift) a cycle of 2π/3(−1, 0, 1) is followed relative to k_y_ encoding.

The blipped‐CAIPI method is as previously described for single‐shot imaging.^16^ However, because a segmented cine sequence is being used, the gradient blips may not necessarily increment linearly in time, but dynamically change depending on the current k_y_ line, and potentially by cardiac phase. Because of the requirement to maintain zero gradient moment within every TR, the additional slice phase increment is removed after each readout. To achieve the desired blipping scheme, the strength of the additional slice gradient is stepped at runtime using the following pattern: *G_blip_* = *G_step_* × [2(*k_y_* mod *f_shift_*) − *f_shift_* + 1], where *G_step_* = *A_blip_*/2/*t_slice_r_*, and *t_slice_r_* is the effective duration of the gradient lobe used for slice re‐phasing. This is done to accommodate the requirement to have an integer step factor at runtime and balance the blipping in k_z_.

For normal cine imaging requirements (parameters detailed in the Methods), the typical largest slice blip required to achieve the greatest inter‐slice shifts is an order of magnitude smaller than the slice refocus area required (in this study, for example, 2.6–8.6% for MB2‐4 with matched shift patterns between slices of FOV/(2–4) and fixed z‐FOV). Therefore, there is only a small overhead on the gradient strength and slew rate, therefore blipping has minimal impact on the minimum TR capability of the sequence. The use of MB pulses, however, will directly impact the minimum TR, particularly at 3T, because of SAR limitations for the typical flip angles used to achieve bright blood contrast.

**Figure 1:**
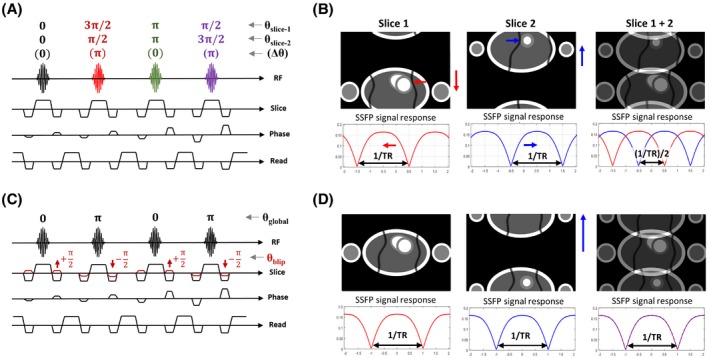
Schematic of RF phase cycled and gradient blipped CAIPIRINHA MB‐bSSFP. (A) Pulse sequence diagram showing the 4‐fold RF phase cycling requirements for a typical MB2 acquisition, alongside (B) the resultant FOV and SSFP frequency response for each slice. (C) Corresponding blipped‐CAIPI MB2 acquisition where a single RF pulse maintains the standard SSFP global phase cycling. Gradient blips (red) are stepped relative to k_y_ position to apply the desired phase alternation to k‐space. The result of using this scheme, illustrated in (D), is that the FOV/2 shift is achieved between slices without any change to the SSFP frequency response, maintaining the original full pass band. For illustration purposes, slice 1 is located at the gradient isocenter

Figure 1 illustrates the main difference between conventional RF phase cycled and blipped CAIPIRINHA MB bSSFP sequences. In this example of MB2, a repeating cycle of 4 pulses is used in the RF phase cycling technique (Figure 1A), whereas in blipped‐CAIPI, a single pulse is used, along with the standard π phase cycling. The slice‐specific phase alternation is achieved by using additional slice gradient blips that cycle dynamically relative to k_y_ position only, and being independent of the RF cycle or cardiac frame position, impose no restriction on TFE factors (Figure 1C). Although both achieve the desired FOV/2 relative shift between slices for the MB2 example shown, the original full pass band of the bSSFP frequency response is only preserved when using the gradient blips. This is indicated pictorially and in the simulation plots of relative signal against off‐resonance angle in Figure 1B,D.

## 3 METHODS

Blipped‐CAIPI MB bSSFP, along with conventional RF phase cycled CAIPIRINHA, was implemented on 2 Philips MR systems (1.5T Ingenia with R5.1.7 software and 3T Tx Achieva with R3.2.2 software). The modified sequence was built around the standard vendor retrospective vectorcardiogram (VCG) gated segmented cine sequence. MB modulation of the vendor default pulse shape (time‐bandwidth product: 2.1) was executed within the scanner pulse sequence code using an analytic function with the parameters required for the desired MB factor and slice separation. MB factors <4 did not use any optimized individual slice phases to reduce peak *B*_1_, whereas MB4 pulses used a static paired slice offset (0‐π‐π‐0) between the inner and outer slices that produces a real‐only RF modulation with peak *B*_1_ reduced from 4× to 3.08× the single‐band equivalent pulse.^20^ Controlled aliasing of MB slices up to FOV/4 shifts were implemented and could be applied independently of the MB factor used. For the conventional RF phase cycled CAIPIRINHA, only relative shifts of FOV/2 with MB2 excitations were tested.

In vivo CMR data were collected from healthy adult volunteers with written informed consent under local ethical guidelines. Overall 5 healthy subjects (4 male, 1 female, age range: 28–35 y) were included in this study, of whom 2 were scanned at 1.5T and 4 at 3T (1 on both). Further details of experiments are given below.

### 3.1 Phantom evaluation of frequency response and eddy currents

Phantom data were acquired to demonstrate the influence of each method on the bSSFP frequency response, emphasized by the addition of an in‐plane linear shim offset to directly visualize the frequency through the spatial locations of dark‐bands. The phantom consisted of an agarose gel (0.5% w/v in normal saline doped with 0.02 mM MnCl_2_) contained in a spherical glass flask, with measured relaxation times: T_2_ ~ 175 ms, T_1_ ~ 2.2s. The phantom was also used to assess the impact additional slice blipping had on the eddy current artefacts observed in bSSFP. All phantom data shown was acquired at 3T using a 32‐channel head coil.

### 3.2 In vivo evaluation of frequency response

In vivo 3T data were collected using a 2 × 16 channel (anterior and posterior element) cardiac coil. Image‐based shimming of the static magnetic field (*B*_0_) was performed in a similar fashion described previously,^12^, ^13^ using *B*_0_ field maps from a multi‐2D SPGR VCG gated sequence acquired in a single breath‐hold at the start of the examination for all 3T exams (parameters: TE = 2.3/4.6 ms, TR = 5.7 ms, acquired resolution = 6 × 6 × 10 mm, 8 slices, scan duration ~20 s). Within each session all bSSFP acquisitions used the same shim settings with comparable exhale breath‐hold position, with only center frequency (f_0_) determination being performed for each.

An exploratory scan on 1 subject (33‐year‐old male, weight: 80 kg) was performed to directly compare conventional RF phase cycled bSSFP with the blipped‐CAIPI equivalent for MB2 (as illustrated in Figure 1) using a reduced stack of 6 slices to compare each MB case from a single breath‐hold (parameters are listed in Table 1, column 1).

**Table 1:** Extended imaging parameters used for in vivo scanning

	3T CAIPIRINHA test		3T fixed‐TR comparison				3T min‐TR comparison				1.5T min‐TR comparison			
Method	RF	blip	blip				blip				blip			
MB factor	2		1	2	3	4	1	2	3	4	1	2	3	4
Resolution (mm)	2 × 2		2 × 2				2 × 2				2 × 2			
Slices	6		12				12				12			
TE (ms)	2		2				1.34	1.57	1.71	1.88	1.55	1.63	1.78	1.83
TR (ms)	4		4				2.7	3.1	3.8	4.2	3.1	3.3	3.6	3.7
Flip angle (°)	45		40				42				54			
Breatholds	1		**6**	**3**	**2**	**1**	**4**	**2**	**2**	**1**	**4**	**2**	**2**	**1**
Time per BH (s)	28.8		18.5	18.5	18.5	27.7	18.0	21.0	16.0	30.0	19.6	22.9	15.3	26.2
Total scan dur. (s)	28.8		110.8	55.4	36.9	27.7	72.0	42.0	32.0	30.0	78.6	45.8	30.5	26.2

A short‐axis stack comprised of 12 slices (with variable slice gap for full ventricle coverage) was acquired in a further 2 subjects (35‐year‐old, 61 kg male and 31‐year‐old, 85 kg male) comparing acceleration factors. Single‐band and blipped‐CAIPI MB 2, 3, and 4 accelerated stacks were acquired using a varying number of breath‐holds. In all cases, acquired resolution was 2 × 2 × 8mm, 30 cardiac phases were reconstructed (minimum 67% sampling), half‐Fourier = 0.63, and TR was fixed to the minimum required for MB = 4 at FA = 40°, to illustrate band locations through MB factors (further parameters listed in Table 1, column 2).

Analysis of g‐factors was performed from these subjects to determine the impact of multiband acceleration and shift pattern. The mean and maximum g‐factor was measured from an ROI covering the entire heart defined using the magnitude bSSFP stack for each acceleration factor on the end‐diastolic frame, with analytic g‐factor maps computed using the method described by Lui et al.^21^

### 3.3 In vivo application of blipped‐CAIPI to LV function assessment

Another subject (28‐year‐old, 85 kg male) was scanned at both 1.5T and 3T using similar parameters except with a flip angle of 54° and 42°, respectively, chosen for optimum blood–myocardium contrast.^12^ The minimum TR achievable for each MB factor was used, permitting the shortest scan time possible for each acquisition and allowing full advantage of reduced susceptibility to banding artefacts. One additional subject (28‐year‐old, 63 kg female) included in supporting information was scanned only with MB4 at 1.5T. The default scanner automated first order volume shimming procedure was used for 1.5T shimming, before the initial stack acquisition without additional operator input, whereas at 3T shimming was as described in the previous section. Full parameters are listed in Table 1, columns 3–4.

An analysis of the SNR in the septum of the myocardium, left and right blood pools, and contrast‐to‐noise ratio (CNR) between the (left and right) blood pool and myocardium was performed using all slices of the stack containing sufficient ROI of all 3 tissues at both end‐systole and end‐diastole. The largest single continuous ellipse that could be drawn for each tissue class was defined on each slice. For the right and left blood pool ROIs, significant trabeculae and papillary muscles were avoided. The SD of the signal in the septum was used as a surrogate measure of the noise SD. An area‐weighted average of all slices was then calculated for end‐systolic and end‐diastolic frames.

For image reconstruction, separate coil sensitivity data was collected either with a standard reference scan—multi‐average (free‐breathing) or single‐average (breath‐hold) ungated low‐resolution 3D rapid SPGR sequence—or by using the time‐average of the single‐band cine stack to provide a matched coil reference. Images were reconstructed both on the scanner using SENSE‐based delayed recon plugin ReconFrame (GyroTools LLC, Zurich) and offline using custom scripts (MATLAB, The MathWorks, Natick, MA) with a hybrid SENSE approach.^22^

## 4 RESULTS

### 4.1 Phantom evaluation of frequency response and eddy currents

**Figure 2:**
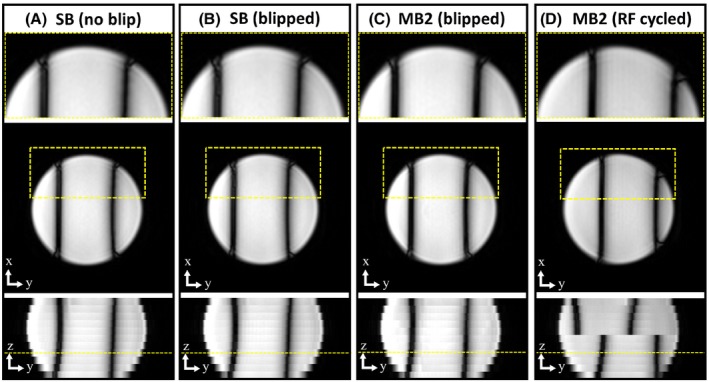
Phantom data comparing single‐band (A), single‐band with blipping (B), alongside MB2 accelerated bSSFP stacks, acquired with blipped (C) and RF phase cycled CAIPIRINHA (D). An additional shim offset (L–R) is applied to visualize the bSSFP frequency response. The lower row shows the image reformatted in the slice direction, highlighting the shift in dark‐bands with RF phase cycling, whereas blipped‐CAIPI MB2 maintains the same frequency response as the single‐band. In (B), the SB is acquired with the same slice blips used in (C) with the displayed in‐plane image (middle row), and zoomed section (top row), being acquired 1× z_gap_ away from the isocenter to expose any increase in artefacts because of eddy currents

The RF phase cycling and blipped methods for controlled aliasing are demonstrated in a phantom (Figure 2) with MB2 accelerated acquisition alongside a single‐band reference. The additional linear *B*_0_ shim offset applied in the left–right direction (as viewed in the figure) after higher order shimming allows for direct visualization of the bSSFP frequency response with spatial location. The locations of 2 dark‐band artefacts revealed in the single‐band reference (Figure 2A) are shifted in equal and opposite directions as a result of the RF phase cycled‐induced (±FOV/4) slice shifts (Figure 2D). Conversely, with blipped‐CAIPI (Figure 2C), slices shift while maintaining standard bSSFP phase cycling leaving the dark‐band artefact in the same location as in the single‐band reference.

The effect on image quality from potential eddy current artefacts arising from the additional alternating slice gradients required for blipped‐CAIPI was assessed by applying the equivalent gradient blips used in MB2 (FOV/2 shift of slices) to a single‐band acquisition (Figure 2B). This was done to separate any artefacts because of slice leakage and associated regional SNR changes correlated to g‐factor of MB folded slices from those of the gradient blips alone. There was no notable impact on image quality between the standard cine (cardiac/phase segmented) acquisition (Figure 2A) and after the addition of slice blipping gradients (Figure 2B).

### 4.2 In vivo evaluation of frequency response

**Figure 3:**
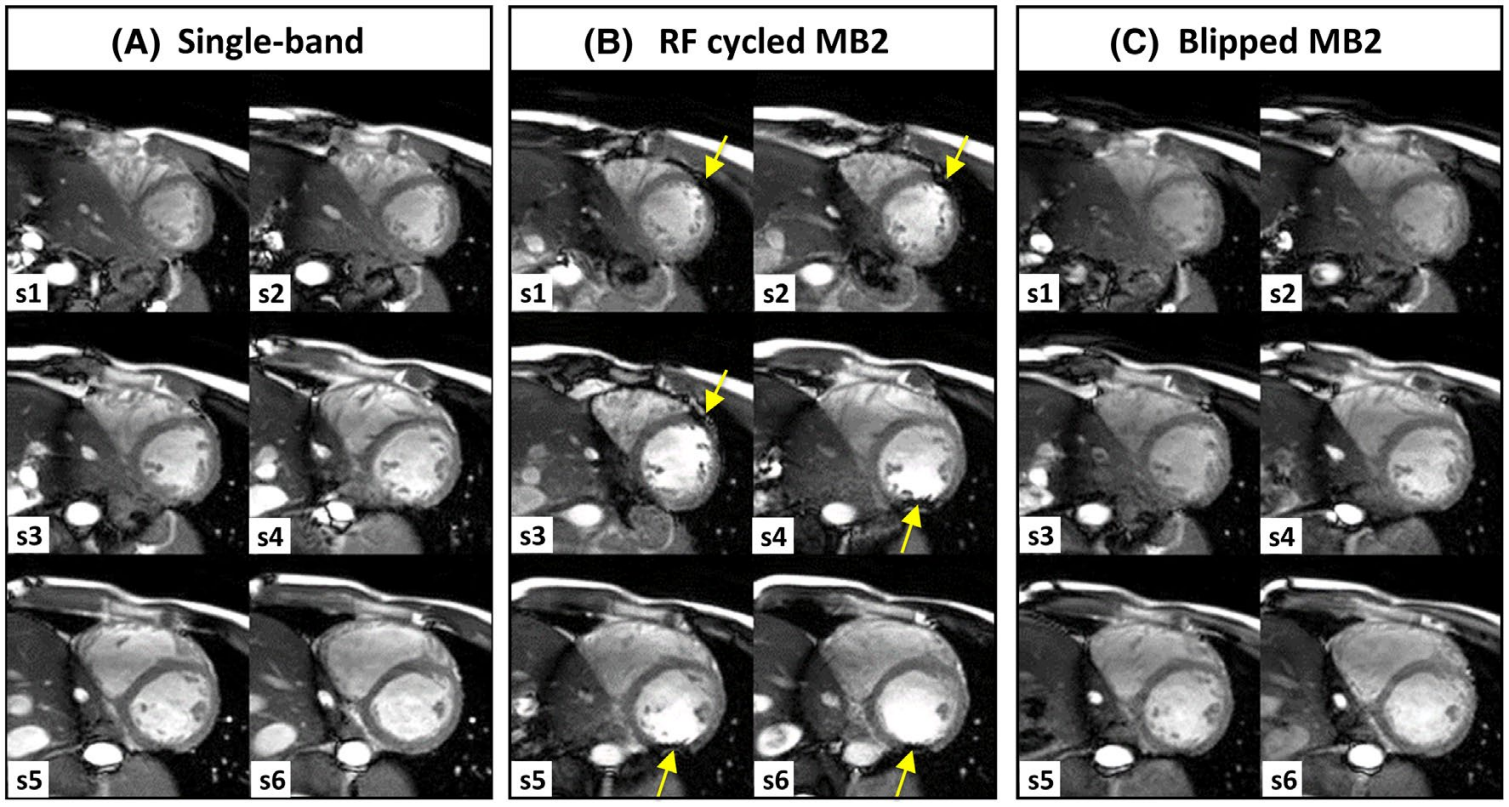
Cardiac cine MB bSSFP data acquired at 3T using image‐based *B*_0_ shimming. Single‐band (A) was acquired in 2 breath‐holds (3 slices each) and MB2 stacks in a single breath‐hold of 6 slices, using both RF cycled (B) and blipped CAIPIRINHA (C). Dark‐band artefacts (yellow arrows) are encroaching on the LV blood pool from alternate sides in the lower and upper halves of the stack with RF cycling, because of the ±FOV/4 shift in MB slices. In blipped‐CAIPI MB2 (C), the same relative slice shift is achieved without altering the bSSFP frequency response, and therefore dark‐band artefacts remain free of the heart region, comparable to single‐band case (A)

MB2 accelerated cardiac cine bSSFP data from a healthy volunteer at 3T using the 2 controlled aliasing methods is shown in Figure 3. Single‐band (2 breath‐holds) and RF phase cycled MB2 and blipped‐CAIPI MB2 stacks acquiring 6 short‐axis slices (single breath‐hold for each MB case) were obtained after initial image‐based shimming using *B*_0_ maps acquired under similar breath‐hold conditions. Although the heart region is clear of dark‐band artefacts for single‐band and blipped‐CAIPI MB2 acquisitions, in RF phase cycled MB2, the location of the bands has shifted with the controlled aliasing of slices as previously described, resulting in dark‐band artefacts encroaching the LV blood pool (yellow arrows) leading to typical unstable signal artefacts.

**Figure 4:**
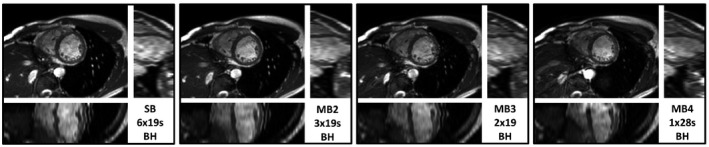
Comparison of single‐band (SB), with blipped‐CAIPI MB2‐4 accelerated cine stacks acquired in a healthy volunteer at 3T. Through‐plane reformats shown below and right, alongside the number of breath‐holds (BH) required to collect each stack. In all cases, the heart remains clear of dark band artefacts with free choice of controlled aliasing being used

A comparison of single‐band, MB2, MB3, and MB4 accelerated (12‐slice) short‐axis cine stacks acquired at 3T using gradient blipping is shown in Figure 4 and Supporting Information Video S1 (cine of mid SA slice). In this test, the TR was fixed at 4 ms for all (the minimum that could be achieved for all MB factors at FA = 40) to directly compare images with matched bSSFP frequency response. Image‐based shimming was used and settings applied to all stacks, only readjustment of f_0_ was performed before each acquisition.

The SNR and blood–myocardium contrast remains sufficiently high across all accelerations to visualize cardiac structures. The through‐plane (long‐axis view) reformats under each mid‐short axis view reveal the benefit of decreasing the number of breath‐holds required, with MB4 allowing for the whole stack to be completed in a single extended (28 s) breath‐hold providing the most consistent alignment of slices. Critically, the banding properties remain the same, and all stacks are free from dark‐band artefacts within the heart lumen, showing that blipped‐CAIPI can be used freely without altering the frequency response. However, the inescapable penalty multiband has on SAR, and therefore minimum TR capability leads to a decrease in the pass band or a restriction of the FA and contrast compared to single‐band. These effects are illustrated in the subsequent section comparing blipped‐CAIPI multiband across acceleration factors and field strength, with minimum TR and optimum FA choice.

**Figure 5:**
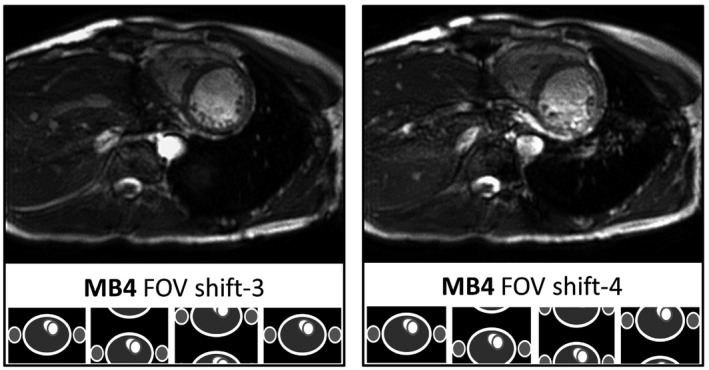
Central slice from 2 blipped‐CAIPI MB4 accelerated cine stacks at 3T, comparing slice shifts of 3 versus 4, with cartoon representations of the controlled aliasing pattern below. Because of the typical short axis orientation and geometry of the coil sensitivities, there is usually a preference for shift‐3 rather than 4, with fewer residual unfolding artefacts present over the region of the heart

The results of the analysis of g‐factor taken from a ROI over the heart in 4 subjects revealed only a modest increase in the average mean and (max) values of: MB2 = 1.00 (1.06), MB3 = 1.06 (1.47), and MB4 = 1.17 (1.61). This suggests that g‐factor noise enhancement with appropriate CAIPIRINHA shifting of slices will not be the limiting factor on multiband acceleration in cardiac bSSFP. Although MB2 acceleration has negligible impact on image quality, residual unfolding artefacts begin to appear with higher MB factors (3–4), predominantly effecting the basal slices. These are more evident while viewing full cine data, because they remain static relative to the dynamic motion of the heart through the cardiac cycle (see Supporting Information Videos S2 [end‐diastolic stack] and S3 [cine of SA slice 11]). The MB2, MB3, and MB4 stacks shown, along with their corresponding g‐factors, used blipped aliasing with a relative shift between MB slices of FOV/2, FOV/3, and FOV/3, respectively. MB4 acquisitions using FOV/4 slice shift generally have stronger unfolding artefacts in the region of the heart, corresponding to the higher local g‐factor (in the case shown in Figure 5, the mean and [max] value increased from 1.13 [2.20] to 1.46 [3.63]). This will vary with typical oblique angles required for conventional cardiac‐oriented views in combination with the geometry of receive elements in the coil being used, along with anatomic variations and general habitus of the subject. Figure 5 illustrates the benefit of being able to select a more favorable shift pattern freely, without having to adjust the number of phase‐encode lines being collected per cardiac phase.

### 4.3 In vivo application of blipped‐CAIPI to LV function assessment

**Figure 6:**
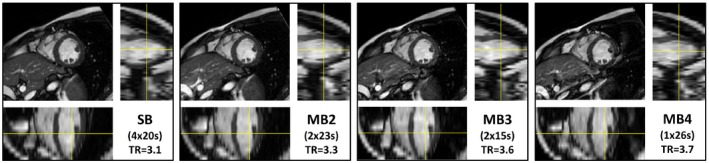
Representative 1.5T bSSFP data comparing single‐band (SB) with blipped‐CAIPI multiband (MB) 2, 3, and 4 accelerated short‐axis stacks. Orthogonal reformats below and alongside show the horizontal and vertical long‐axis views through the stack. The inset label documents the breath‐holds required to complete each stack in brackets, and the TR in milliseconds achieved for each sequence (full cine version in Supporting Information Video S5)

The blipped‐CAIPI method was also tested on a 1.5T system comparing full cine stacks acquired with up to MB4 acceleration, which again allowed for a full stack being acquired in a single extended breath‐hold (Figure 6). Here, the performance of MB acceleration in cine bSSFP is potentially more advantageous because of SAR limitations being less restrictive. In the example shown, the optimum flip angle for 1.5T (54°)^12^ was used with a TR ≤3.7 ms up to MB4, resulting in higher contrast between blood and myocardium. In this example, each MB case also used the minimum TR available to offer a full and fair comparison across stacks. The influence MB has on minimum TR (3.1 to 3.7 ms), results in overall scan times in the example shown of 4 × 20, 2 × 23, 2 × 15, and 1 × 26 s breath‐holds for MB1–MB4, respectively.

**Figure 7:**
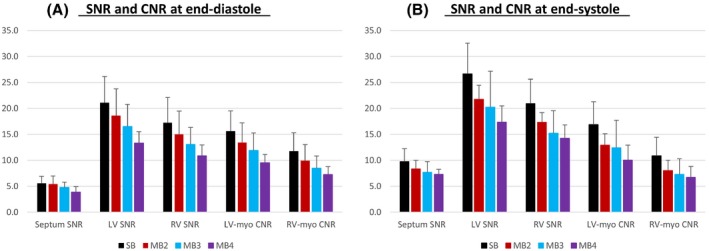
Mean SNR in the septum, left (LV) and right (RV) ventricular blood, and CNR between blood and myocardium (LV‐myo and RV‐myo) across multiband factors at 1.5T at end‐diastole (A) and end‐systole (B). The weighted mean (+SD) values across the short‐axis stacks are from the subject shown in Figure 6. The accumulated scan duration for each single‐band (SB) and multiband (MB) 2‐4 stack was 78.6, 45.8, 30.5, and 26.2 s, respectively

The SNR, and importantly, blood–myocardium contrast measured across the heart, remains high through the MB factors used (Figure 7). There is a proportional decline in SNR with MB factor, although this is primarily in the blood pool and exceeding the associated increase in mean g‐factor alone. The likely reason is because of a reduction of in‐flow signal enhancement because of saturation of simultaneous slices. However, when considering the overall reduction in scan time, there is a net boost in signal per unit time ($SNR/\sqrt{\mathrm{TA}}$), peaking at MB3.

Leakage artefacts at 1.5T are less problematic than at 3T and are mainly evident in MB4 reconstructions when viewing full cine stacks (Supporting Information Video S4). The same subject was also scanned at 3T using a similar minimum TR MB comparison, however, the SAR penalty resulted in a more rapid rise in TR despite a lower optimum FA of 42° being used. In this case, the TR rose from 2.7 ms to 4.2 ms (MB1–MB4), and although the shimming was carefully controlled so that bands did not land in the blood pool, not all the myocardium was free of bands in all slices for this subject. Notably, the leakage artefacts for the same subject were also noticeably worse at 3T (see Supporting Information Video S6). An additional subject scanned at 1.5T with only MB4 (single 24‐s breath‐hold) is included in Supporting Information Video S7, providing further evidence that good contrast and low (leakage) artefact level can be achieved more readily at lower field.

The increase in RF pulse amplitude with higher MB factors did not lead to the peak *B*_1_ hardware limits (13.5 μT) being reached in any case tested at 3T (with the largest being 10.14 μT for MB4). Instead, SAR was the overriding constraint with minimum TR for all MB factors. However, at 1.5T, the peak *B*_1_ amplitude of 20 μT was reached for both MB2–MB3, and the minimum TR was achieved within SAR constraints. For MB4, using the individual slice phase combination to reduce peak amplitude meant the SAR limit was reached before peak *B*_1_, whereas, without the use of this phase combination, peak *B*_1_ would have been reached first and the minimum TR extended from 3.7 to 3.9 ms.

## 5 DISCUSSION

In this study, we have demonstrated the potential of using blipped‐CAIPI MB bSSFP for cardiac imaging, with slice accelerations up to 4, enabling full short axis stacks to be acquired within a single (extended) breath‐hold. The technique was implemented into a standard retrospectively gated segmented cine sequence typically used to assess cardiac function. Data were collected in phantoms and in vivo at both 1.5 and 3T field strengths, along with comparative MB2 acquisition using the RF phase cycled technique. The principal advantage of using blipped‐CAIPI is preserving the bSSFP frequency response, which allows it to be more easily applied at higher field strengths without increasing the problematic effects of dark‐band artefacts, compared to using traditional RF phase cycled CAIPIRINHA. Data presented (Figure 3) comparing the 2 methods of controlled aliasing revealed that despite use of second order image‐based *B*_0_ shimming, it is typically not feasible at 3T to avoid dark‐band artefacts impinging on the heart when using the conventional RF phase cycling approach, where slices are shifted equally by ¼ of the FOV. The recent development of applying an unbalancing gradient to adjust the local Larmor frequency^15^ addresses this limitation (although with some signal loss because of imposed through‐slice dephasing) and therefore, in general, should allow both methods to achieve similar MB accelerations.

There are several additional differences to consider between each of the methods used for controlled aliasing in MB imaging. Apart from the primary issue of altering the bSSFP frequency response, the RF phase cycling method requires increasingly large sets of pulses to create the desired slice shifts required to limit g‐factors at higher MB accelerations. This imposes additional restrictions on the desired parameter choices, because of the requirement to complete the RF phase cycle within each cardiac phase and k_y_ segment, whereas the gradient blipping method allows freedom to use the optimum number of lines per cardiac segment, with only a single MB pulse and conventional bSSFP phase cycling being used. Without the restriction on changing individual slice phases dynamically, the use of optimum static slice phases to reduce the RF peak *B*_1_ is also straightforward to apply, which was shown to directly benefit the minimum TR capability in this study for MB4 at 1.5T.

Blipped‐CAIPI as implemented in this study allows for free selection of the optimum slice shift pattern that can noticeably improve reconstruction (Figure 5). This preferred aliasing pattern may change between subjects, because it is dependent on the rotations required to achieve the specified cardiac‐oriented views in relation to the coil geometry. As with in‐plane acceleration, the coil sensitivities along the direction of aliasing (usually directly determined by the orientation of the imaging plane), along with the required phase‐encode FOV, will ultimately limit the maximum MB acceleration, within acceptable image quality. In general, MB acceleration is preferable to in‐plane acceleration as the SNR loss depends on g‐factor alone without the associated $\sqrt{R}$, where *R* is the acceleration factor, loss from subsampling k‐space lines.^2^

MB acceleration does, however, come with the disadvantage of increasing the energy of the RF pulse, and hence the SAR burden of the sequence, directly with increasing MB factor. This can partly be mitigated by a combination of stretching the pulse and using optimum static slice phases to reduce peak *B*_1_, however, this impacts on the minimum TR and/or maximum FA achieved. Therefore, there will be diminishing returns from using very high MB acceleration, and this was the reason for limiting to MB4 in this study. Nevertheless, as demonstrated, this could lead to an effective overall scan time reduction and even allow for a complete stack being acquired within a single breath‐hold. Because of the SAR dependence with the field strength, it is more advantageous to apply the technique at lower fields, as demonstrated in this study comparing the same subject at both 1.5T and 3T. Here, MB2‐4 was used with the higher optimum flip angle suggested for 1.5T, yet still achieved a shorter TR, resulting in higher contrast compared to 3T data. All MB factors tested were SAR limited at 3T, whereas at 1.5T, SAR was the overriding constraint only for MB4, which was partly because of use of a slice phase combination scheme to reduce peak *B*_1_ for this pulse, allowing for a reduced TR. More advanced pulse design and sequence optimization methods that reduce the SAR of multiband pulses have recently been demonstrated^23^. This includes the use of VERSE with optimal phase combination to achieve minimum TR, which could improve the applicability of MB bSSFP at 3T.

In addition, the scanners used in this study enforced long‐term (i.e., 6 min) SAR limits and also included restrictions on local (i.e., 10 g averaged) SAR. The 2015 IEC guidelines (60601‐2‐33:2015) do not require control of local SAR for volume transmit coils. They also allow for a doubling of the SAR limit for short scans (the limit can be doubled in any 10‐s period), which would be relevant for the short breath‐hold scans used in this study. Hence, it would be possible for significantly relaxed SAR limits to be applied. This would greatly benefit MB bSSFP at 3T, where local SAR was the limiting factor, by reducing TR and lessening the sensitivity to off‐resonance artefacts and shortening breath‐holds, or increasing the flip angle for better contrast.

A further observation from this study is that the reconstruction of data collected at 3T suffered from increased artefacts compared to 1.5T. In addition to the issues of field homogeneity being more challenging at 3T, and therefore dark‐bands being more problematic, the residual slice leakage of abdominal fat appears more problematic. Although some of this may be related to breath‐hold differences between accelerated data and collection of coil reference data, another factor could be the relative water–fat shift being greater with field strength. The limited bandwidth of the RF pulse will result in a small mismatch in the slice excitation band of the fat and water, which will increase as the RF pulse is stretched because of peak *B*_1_ or SAR limitations. The leakage artefact problem may also be tackled during reconstruction, therefore this could be an area to investigate for improving cine MB bSSFP where fat saturation cannot be readily used without breaking the steady state.

The additional gradient that alternates between successive TRs required for blipped‐CAIPI changes the inherent flow and eddy‐current sensitivity, which could lead to increased artefacts. However, our experiments using phantoms (Figure 2) did not reveal any discernible difference in image quality as tested on single‐band acquisitions with, and without, the slice blips required for controlled aliasing. Although the effect of eddy‐currents will be system‐dependent, one explanation for the benign effect observed in this study is that the change in gradient area typically required between TRs is much smaller than that of the existing phase‐encoding gradient jumps between cardiac phases for typical Cartesian segmented cine acquisitions, with typical parameters used in 2D stack acquisitions.

Although not directly assessed in this study, the addition of slice blips should also be negligible in relation to the flow compensation properties of the bSSFP sequence. There was little evidence of extra flow artefact observed in vivo compared to single‐band, where the dominant artefacts from high MB acceleration were static in time (Supporting Information Videos [Link], [Link], [Link]–[Link], [Link], [Link]). In addition, although flow compensation is traditionally maintained over the TR period in the measurement and slice directions, it is unlikely that typical cine bSSFP sequences used for functional CMR assessment are flow‐compensated at the TE, because the additional bipolar gradient required for this adds significant overhead on the minimum TR. Consequently, the main change from standard operation is that blipped‐CAIPI MB bSSFP changes the flow compensation slightly in the slice direction, caused by the use of a simple additional gradient step around the existing gradient re‐winder and dephasing objects. This is typically of the order equivalent to that incurred by jumping by 1 to 3 k_y_ phase encode lines (relative to slice blips required for FOV/2 slice shift at 60 mm separation through to FOV/3 at slice separation of 30 mm), again less than already typically applied in phase‐encode jumps between each cardiac phase in segmented cine acquisitions.

The addition of slice gradients in blipped‐CAIPI, if applied using a balanced pattern, does not cause any inherent signal loss because of dephasing. It can be considered analogous to phase encoding in the slice direction as used in 3D imaging and the reconstruction formulated as an undersampling in the k_y_‐k_z_ plane as described in Zahneisen et al.^24^ However, other factors that do have an impact on the measured SNR and contrast with increasing MB acceleration are likely because of the saturation of inflowing blood from neighboring slices. This effect should be limited with bSSFP because of inherent T_2_/T_1_ contrast, compared for example, to MB accelerating of cine FLASH that more heavily relies on inflow enhancement for good contrast.

One limitation with using higher MB factors, as with all parallel imaging‐based acceleration, arises from SENSE reconstruction artefacts. For cardiac and abdominal imaging, however, this is also influenced by the way in which the coil reference is acquired. Any significant mismatch of the coil position between the reference—sometimes acquired free‐breathing—and the accelerated breath‐hold scan that may push the anterior receive coil to a different position, could contribute to increased slice leakage artefacts. Alternatively, as demonstrated in recent studies using MB‐accelerated SPGR acquisition, auto‐calibrated^4^, ^5^ data should create the most consistent coil reference possible and also eliminate the need for a separate reference scan. Combining this approach with retrospective gating, however, will lead to a more complex reconstruction problem, because the MB unfolding and temporal interpolation of missing lines of data cannot be treated separately. All data shown here were reconstructed using spatial parallel imaging methods independently frame‐by‐frame, therefore fully preserving temporal resolution. Nonetheless, future developments could consider self‐calibrated approaches and potentially trading off temporal regularization and artefact reduction. Finally, although not explored in this study, combining in‐plane and slice acceleration could offer the potential to further reduce breath‐hold durations given that ample SNR and contrast has been demonstrated for MB4 acceleration at 1.5T.

## 6 CONCLUSION

We present the use of blipped‐CAIPI MB bSSFP cine imaging, with slice accelerations of up to 4, enabling functional volumetric coverage of the heart within a single breath‐hold. The method is fully compatible with standard retrospective‐gated cine sequences allowing for optimal acceleration designs unhindered and, most importantly, does not alter the bSSFP frequency response. Because of the inevitable increase in SAR and peak *B*_1_ of pulses with multiband factor, the method is more effective at lower field, although MB2 can be readily applied across subjects at 3T with minimal impact on image quality because of TR increase or reconstruction artefacts.

## Supporting information


**VIDEO S1** In vivo data from 3T (fixed TR) blipped‐CAIPI MB bSSFP showing cine of mid short axis slice with slice accelerations 1–4

**VIDEO S2** In vivo data from 3T (fixed TR) blipped‐CAIPI MB bSSFP showing full short axis stack at end‐diastole with slice accelerations 1–4

**VIDEO S3** In vivo data from 3T (fixed TR) blipped‐CAIPI MB bSSFP showing cine of the basal slice most effected by leakage artefacts at high MB factor

**VIDEO S4** In vivo data from 1.5T (minimum TR) showing the full short axis cine stack comparison of blipped‐CAIPI MB bSSFP using slice accelerations of 1–4

**VIDEO S5** In vivo data from 1.5T (minimum TR) showing multi‐planar reformats (movie version of Figure 6) comparing blipped‐CAIPI MB bSSFP using slice accelerations of 1–4

**VIDEO S6** In vivo data from 3T (minimum TR) showing full short cine stack comparison of blipped‐CAIPI MB bSSFP using slice acceleration 1–4, with the same subject as used in Videos S4‐5 in order to compare performance across field strengths

**VIDEO S7** Additional in vivo data from 1.5T (minimum TR) showing full short cine stack of blipped‐CAIPI MB4 only, acquired in a single 24‐s breath‐hold
