# Supplementary figures and images for: Simultaneous multislice imaging of the heart using multiband balanced SSFP with blipped‐CAIPI
Source: Magn Reson Med. 2019 Nov 20;83(6):2185–96. doi: 10.1002/mrm.28086 (PMC7065016; doi:10.1002/mrm.28086)

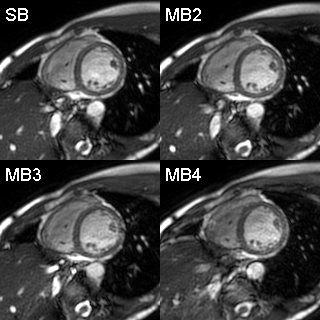

Supplement: Supplementary file 1 — VIDEO S1 In vivo data from 3T (fixed TR) blipped‐CAIPI MB bSSFP showing cine of mid short axis slice with slice accelerations 1–4 [file MRM-83-2185-s001.gif]

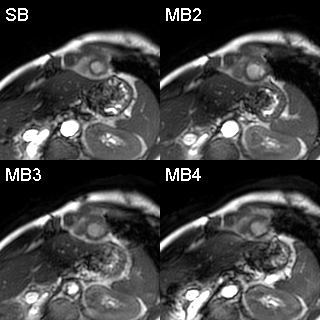

Supplement: Supplementary file 2 — VIDEO S2 In vivo data from 3T (fixed TR) blipped‐CAIPI MB bSSFP showing full short axis stack at end‐diastole with slice accelerations 1–4 [file MRM-83-2185-s002.gif]

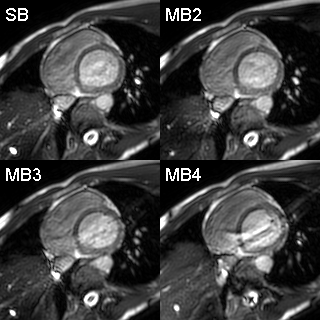

Supplement: Supplementary file 3 — VIDEO S3 In vivo data from 3T (fixed TR) blipped‐CAIPI MB bSSFP showing cine of the basal slice most effected by leakage artefacts at high MB factor [file MRM-83-2185-s003.gif]

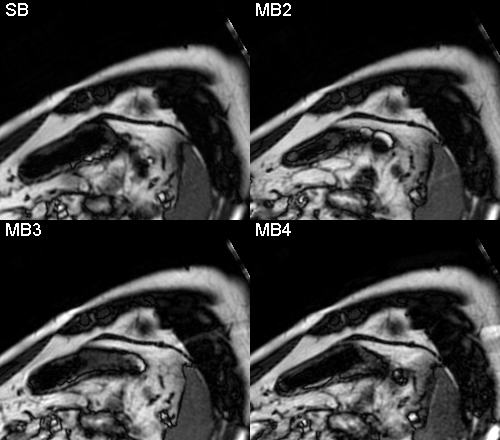

Supplement: Supplementary file 4 — VIDEO S4 In vivo data from 1.5T (minimum TR) showing the full short axis cine stack comparison of blipped‐CAIPI MB bSSFP using slice accelerations of 1–4 [file MRM-83-2185-s004.gif]

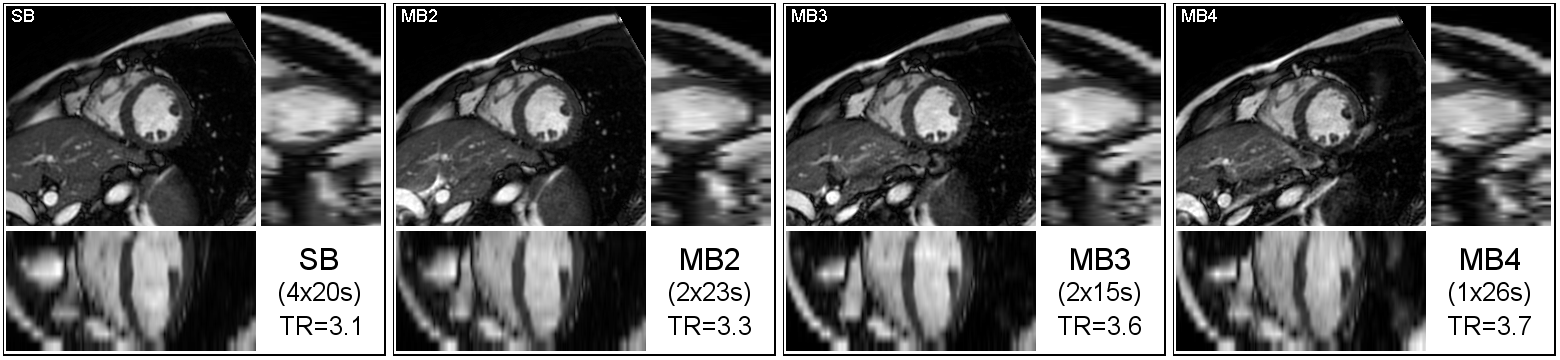

Supplement: Supplementary file 5 — VIDEO S5 In vivo data from 1.5T (minimum TR) showing multi‐planar reformats (movie version of Figure 6) comparing blipped‐CAIPI MB bSSFP using slice accelerations of 1–4 [file MRM-83-2185-s005.gif]

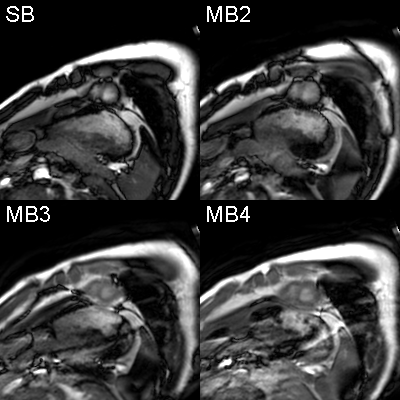

Supplement: Supplementary file 6 — VIDEO S6 In vivo data from 3T (minimum TR) showing full short cine stack comparison of blipped‐CAIPI MB bSSFP using slice acceleration 1–4, with the same subject as used in Videos S4‐5 in order to compare performance across field strengths [file MRM-83-2185-s006.gif]

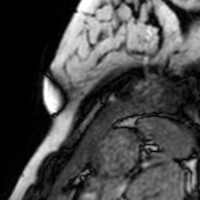

Supplement: Supplementary file 7 — VIDEO S7 Additional in vivo data from 1.5T (minimum TR) showing full short cine stack of blipped‐CAIPI MB4 only, acquired in a single 24‐s breath‐hold [file MRM-83-2185-s007.gif]
